# Upregulation of miR-183 inhibits the invasion and migration of endometrial stromal cells in endometriosis patients by downregulating Ezrin

**DOI:** 10.3389/fonc.2025.1537528

**Published:** 2025-06-24

**Authors:** Jinmei Shi, Junbo Cai, Lin Kong, Lingxiao Ying, Xing Liu, Mengting Jiang, Dan Pan

**Affiliations:** ^1^ Department of Obstetrics and Gynecology, Taizhou Municipal Hospital Affiliated with Taizhou University, Taizhou, Zhejiang, China; ^2^ Department of Surgical Oncology, Taizhou Municipal Hospital Affiliated with Taizhou University, Taizhou, Zhejiang, China; ^3^ Department of Obstetrics and Gynecology, Hangzhou Women’s Hospital, Hangzhou, Zhejiang, China

**Keywords:** microRNA-183, Ezrin, endometriosis (EM), ectopic endometrial stromal cell (ESC), migration

## Abstract

**Purpose:**

The present study investigated the expression and role of miR-183 in the proliferation, invasion, migration, and apoptosis of endometrial stromal cells in endometriosis patients and the potential involvement of targeting Ezrin.

**Methods:**

Normal, non-ectopic, and ectopic endometrial stromal cells (ESCs) were extracted from endometrial samples. RT-qPCR was used to evaluate miR-183 expression levels in endometrial tissue samples. Flow cytometry, cell proliferation assay, adhesion assay and Transwell assays and cell scratch assay were performed to assess cell apoptosis, viability, migration, and invasion of cells transfected with miR-183 inhibitor, miR-183 mimics, or controls. Western blotting was used to determine the expression of the migration-and invasion-related proteins. The expression status of RhoA/ROCK/Ezrin in endometriosis was verified by animal models.

**Results:**

miR-183 expression levels were markedly downregulated and RhoA and Ezrin expression levels were upregulated in ectopic endometrial samples. Upregulation of miR-183 expression inhibited cell apoptosis, migration and invasion and promoted cell adhesion in ESCs, but had no significant impact on cell proliferation. miR-183 mimics decreased the expressions of Ezrin, RhoA, RhoC, and Rock.

**Conclusion:**

Upregulated expression of miR-183 promoted cell adhesion and suppressed the apoptosis, invasion, and migration of ESCs by downregulating Ezrin. miR-183 may play a suppressor role in endometriosis by downregulating Ezrin to inactivate the Rho/ROCK pathway.

## Introduction

Endometriosis (EM) is a common gynecological endocrine condition characterized by the abnormal presence of endometrial stroma and glands outside the uterus. EM most commonly affects organs of the pelvic cavity, including ovaries, fallopian tubes, urinary bladder, peritoneum, and intestines (1, 2). In rare cases, EM is located in other organs beyond the pelvis, such as the pleura, diaphragm, abdominal wall, and the central or peripheral nervous system (1, 2). The World Health Organization reported that the incidence of EM in reproductive-aged women globally is approximately 10% (3). Most patients with endometriosis are between 25 and 45 years (4). Endometriosis has a diverse array of clinical symptoms, ranging from accidentally found asymptomatic lesions to severe conditions, and the symptoms do not depend on the lesion size (4). The main symptoms caused by endometriosis include dysmenorrhea, dyspareunia, pelvic pain, infertility, abundant irregular menstruation, back pain, gastrointestinal problems, and mental stress, which affect a woman’s overall health as well as her psychological and social well-being and significantly reduces quality of life (5–9).

The pathogenesis of EM is currently unclear, and none of the current theories can fully explain the causes of EM pathogenesis. Multiple factors such as the hormone environment, immunologic derangement, genetic predisposition, and lifestyle factors are believed to be potential causes for the development of endometriosis (10). While EM is classified as a benign gynecological disorder, it shows malignant biological behaviors such as distant metastasis, invasion, uncontrolled cell proliferation, adhesion, implantation, and recurrence (11). There is growing evidence that changes in ESC behaviors, such as proliferation, migration, and invasion, can promote the onset and development of EM (12–14).

MicroRNAs (miRNAs) are small noncoding RNAs with a length of 20–24 nucleotides that participate in RNA silencing and the post-transcriptional regulation of gene expression (15–17). MiRNAs are involved in a variety of cellular biological behaviors, including cell differentiation, proliferation, migration, invasion, and apoptosis (18, 19). The alteration of miRNA expression can cause abnormal expression of many genes, thereby significantly affecting cell functions. Increasing evidence has suggested that miRNAs are involved in the development of various human diseases, including cancer, cardiovascular disorders, metabolic diseases, and reproductive diseases (20–23). Several reports showed that the deregulation of miRNA expression is associated with the pathogenesis of endometriosis (24–26). Therefore, better understanding of the differential miRNA expression profiles in EM may provide insights into EM pathogenesis (27–29).

miR-183 is one of the most widely studied miRNAs. Previous studies have shown that miR-183 is abnormally expressed in multiple tumors (30, 31), showing tumor-suppressive or oncogenic functions, depending on the tissue type. Previous studies have revealed that miR-183 contributes to causing apoptosis in endometrial stromal cells and negatively regulates cell invasiveness (32, 33). However, the specific regulatory mechanism has not been investigated thus far.

Ezrin is a member of the Ezrin/radixin/moesin family and a membrane-cytoskeleton linker protein. Several studies showed that Ezrin functions in the metastasis and invasion of tumors, including breast cancer, colorectal carcinoma, thyroid cancer, pancreatic ductal adenocarcinoma, gastric cancer, and serous ovarian carcinoma (34–36). High-throughput miRNA chip detection combined with bioinformatics tools showed that miR-183 regulates Ezrin protein expression, and additional analyses showed that miR-183 participates in cell infiltration and migration by regulating Ezrin protein expression (37). The Rho/ROCK signaling pathway plays an important role in cell biology, especially in regulating cell contraction force, reconstruction of the cytoskeleton, and signal transmission. Moreover, activation of the Rho/ROCK signaling pathway also plays an important role in the development of endometriosis and is closely related to ezrin. Activation of the Rho/ROCK pathway not only promotes the activation of ezrin, but also further affects the remodeling of the cytoskeleton, thereby regulating the cell’s adhesion, migration and invasion capabilities. This signaling pathway plays a key role in promoting the growth of ectopic endometrial stromal cells and anti-apoptotic, thus promoting the development of endometriosis.

In the present study, we investigated the expression levels of miR-183 in endometriosis patients. We further explored whether miR-183 expression influences the malignant behaviors of endometrial stromal cells in endometriosis patients by targeting Ezrin.

## Materials and methods

### Patient population

A total of 20 paired ectopic and eutopic endometrial tissues were collected from patients with ovarian endometriotic cysts and normal endometrial tissues were collected from 50 patients with other benign ovarian cysts from January 2022 to April 2025. Ovarian specimens were obtained through laparoscopy, while endometrial specimens were obtained under hysteroscopic monitoring. All tissues were from Taizhou Municipal Hospital. The patients’ ages ranged from 26 to 54 years. The inclusion criteria were as follows: 1) patients treated at Linyi Central Hospital for the first time without hormone therapy prior to laparoscopic surgery. 2) patients who were diagnosed laparoscopically and histologically with American Fertility Association (AFS) revised standard staging score (the RAS score) stage III/IV endometriosis. 3) patients had regular menstrual cycles (21–35 days). The exclusion criteria were as follows: 1) patients who did not undergo surgery. 2) patients had taken any combined hormonal contraception ahead of operation. 3) patients had other surgical, endocrine, immune, or metabolic disease. All samples were collected at the time of surgery, immediately frozen in liquid nitrogen, and stored at -80°C. The specimen collection protocol was approved by the Ethics Committee of Taizhou Municipal Hospital of Zhejiang, and all patients provided signed informed consent.

### Animals and mouse model of endometriosis

Mature female C57BL/6 mice (7 weeks-old) were purchased from Shanghai Model Organisms Center, Inc. (Shanghai, China) and were allowed to acclimate for 1 week before surgery. One week before modeling, diethylstilbestrol (0.1 mg/kg) was subcutaneously injected into the neck and back of mice (including donor mice and recipient mice), once every three days for a total of two injections to ensure that the mice were uniformly in the estrus period during the operation. From the injection of estrogen until the day of intraperitoneal implantation, vaginal exfoliated cell smear examinations were conducted on donor mice and recipient mice at regular intervals every day to observe the estrous cycle of the mice. Mice were intraperitoneally anesthetized with 1% pentobarbital sodium (5 ml/kg). The uterus of the donor mouse was carefully harvested. Subsequently, the harvested uterus was meticulously rinsed with pre - cooled normal saline to remove residual blood and mucus. The uterus was then bisected and separately placed in petri dishes filled with pre-cooled RPMI1640 medium. Using ophthalmic scissors, the uterus was longitudinally incised. The endometrial layer was carefully peeled off, and promptly minced into 1-mm³ fragments. These endometrial fragments were then suspended in an appropriate medium, ready for subsequent use. The endometrial fragments were thoroughly mixed with Matrigel (Cat. 354230, Corning, New York, NY, USA) at a 3:1 volume ratio. Prior to transplantation, the lateral abdominal skin of recipient mice was carefully shaved and disinfected with an appropriate antiseptic solution. Using a sterile syringe, the endometrial-Matrigel mixture was precisely injected into the lateral abdominal wall of each recipient mouse. Immediately after injection, a small amount of penicillin was applied topically to the injection site to prevent potential infections. For the sham operation group, the injection procedure was identical to that of the model group. Instead of endometrial fragments, an equivalent volume of abdominal adipose tissue harvested from donor mice was injected, serving as a negative control. On the day of the surgical procedure, recipient mice were administered a subcutaneous injection of β-estradiol benzoate solution at a dosage of 150 μg/kg body weight. This hormonal administration regimen was executed with a dosing interval of four days, and a total of two injections were administered to each animal. 21 days pos-transplantation, after confirming deep anesthesia, the mice were euthanized. Subsequently, an examination was conducted to observe the morphological characteristics of the ectopic endometrium in the model group, including coloration, texture, and vascular patterns. The volume of the grafts was measured. All the animal procedures were performed in accordance with legal requirements and under licensed approval from the Ethics Committee of Taizhou Municipal Hospital of Zhejiang.

### Cell culture and cell transfection

Tissue samples were dissected, washed three times with PBS, and digested with collagenase II and 0.25% trypsin at 37°C for 45 min. Ectopic endometrial stromal cells (ectopic EM cells) were isolated from peritoneal endometriotic lesions of patients with confirmed endometriosis. Eutopic endometrial stromal cells (Eutopic EM cells) were obtained from eutopic endometrium of the same patients. Eutopic endometrial stromal cells (Eutopic normal cell) were derived from disease-free individuals undergoing benign gynecologic surgeries. These cells cultured in DMEM (Gibco; Thermo Fisher Scientific, Inc, Waltham, MA, USA) supplemented with 10% FBS (Gibco; Thermo Fisher Scientific, Inc.) at 37°C in 5% CO2 for 24 h. Control oligo, hsa-miR-183, si-RhoA (5’-GAAGUCAAGCAUUUCUGUC-3’) and si-Ezrin (5’-ACCAATCAATGTCCGAGTTACC-3’) were chemically synthesized by GenePharma (Shanghai GenePharma Co., Ltd, Shanghai, PR China).

The cells were divided into the following groups: the eutopic norm group, the eutopic EM group, the ectopic EM group, the mimics control group, the miR-183 mimic group (transfected with miR-183 mimics), the inhibitor control group, and the miR-183 inhibitor group (transfected with miR-183 inhibitors). The Lipofectamine 3000 transfection kit (Thermo Fisher Scientific, Inc.) was used for cell transfection following the manufacturer’s protocol. At 48 h post transfection, cells were collected for further experiments.

### Reverse transcription–quantitative polymerase chain reaction analysis

Total RNA was extracted by the EZ-10 Kit (Shenggong Biotechnology Co., Ltd, Shanghai, China) and reverse transcribed to cDNA using a reverse transcription kit (Cowin Biotech Co., Ltd, Beijing, China) in accordance with the manufacturer’s instructions. qPCR (SYBR Green qPCR kit, Yeasen Biotechnology (Shanghai) Co., Ltd, Shanghai, China) was performed XXX. (Roche LightCycler 96 qPCR Real Time PCR system, F. Hoffmann-La Roche, Ltd, Basel, Switzerland). The amplification conditions were as follows: pre-denaturation at 95°C for 10 min, denaturation at 95°C for 15 s and annealing at 60°C for 60 s (for 40 cycles). U6 was used as an internal reference for miR-183. The expression levels of the target genes were calculated by the 2-ΔΔCq method. Each experiment was repeated three times and the average value was calculated. The primers used were: miR-183-F, TATGGCACTGGTAGAATTCACT; miR-183-R, GCGAGCACAGAATTAATACGAC; U6-F, CGCTTCGGCAGCACATATACTA; U6-R, CGCTTCACGAATTTGCGTGTCA; Mouse Ezrin-F, ATCGAGGTGCAGCAGATGAAGG, Mouse Ezrin-R, CGGAGCATCTGCTCCTTTTCTC; Mouse RhoA-F, CTTCAGCAAGGACCAGTTCCCA, Mouse RhoA-R, GGCGGTCATAATCTTCCTGTCC; Mouse RhoC-F, GAGGCAAGATGAGCATACCAGG, Mouse RhoC-R, GCCATCTCAAATACCTCCCGCA; Mouse ROCK1-F, CACGCCTAACTGACAAGCACCA, Mouse ROCK1-R, CAGGTCAACATCTAGCATGGAAC; Mouse GAPDH-F, CATCACTGCCACCCAGAAGACTG, Mouse GAPDH-R, ATGCCAGTGAGCTTCCCGTTCAG.

### Transwell migration and invasion assays

For migration assays, uncoated chambers were used. For invasion assays, Matrigel was thawed at 4°C and diluted in serum-free DMEM at 1:3; the solution (30 µL) was added into the top chamber of Transwell chambers (Corning Incorporated, New York, USA) and allowed to solidify. Transfected cells were inoculated in the top chambers in serum-free medium and medium containing serum was included in the bottom chamber; plates were cultured at 37°C and 5% CO2 for 24 h (migration assay) and 48h (invasion assays). Cells in the chamber were washed gently with PBS and stained with crystal violet staining solution(Shanghai Qiangshun Chemical Reagent Co., Ltd, Shanghai, China) at room temperature for 30 min. Cells in five randomly selected fields were evaluated; the cells were photographed using light microscopy at x200 magnification. For quantitative analysis, the stained cells were dissolved with 300 µL of 30% acetic acid. The supernatant was then collected, and the optical density (OD) was measured at 570 nm using a microplate reader. The experiments were repeated three times and the results were averaged.

### Scratch assay

Cells were cultured in 6-well plates with DMEM supplemented with 10% fetal bovine serum (FBS) and incubated at 37°C under 5% CO_2_ until confluent monolayers formed. A uniform scratch was generated in each monolayer using a sterile 200 µL pipette tip, held vertically and dragged gently across the well surface in a straight line. After scratching, detached cells and debris were removed by washing twice with phosphate-buffered saline (PBS), and fresh serum-free medium was added to restrict proliferation. The plates were immediately transferred to an environmental chamber (37°C, 5% CO_2_). Cell migration into the wound area was monitored over 48 hours, with images acquired at 0 and 48 h post-scratch. Three independent experiments, each with triplicate wells, were analyzed, and statistical significance was determined using Student’s t-test (*P* < 0.05).

### Adhesion assay

Coating buffer (50–100 µL) was seeded in 96-well flat-bottom culture plates (Thermo Fisher Scientific, Inc.), and the plates were incubated overnight at 2°C to 8°C. The cells were seeded in each well of a 96-well-plate at a density of 5×105 cells/well and the plates were incubated at 37 °C in a 5% CO2 incubator for 2 h. After washing in PBS, cells were stained by Cell Stain Solution B (Cell Adhesion Assay Kit, Beibo Biotechnology Co., Ltd, Shanghai, China). The optical density of the sample was measured at 450 nm. Adhesion ratio = [(treatment group OD value/BSA group OD value) − 1] × 100.

### Cell proliferation assay

Cells were seeded into 96-well plates, and the medium was changed after 12 h. Five wells were prepared for each experimental group. The medium was replaced after 48 h.CCK-8 (Cell Counting Kit-8, Beyotime Institute of Biotechnology, Shanghai, China) solution was added and the absorbance value was determined with a microplate reader. The assay was repeated three times and the results were averaged.

### Apoptosis analysis

Cells were plated in a 6-well plate (1.2×106 cells/well). Next, 500 µL Binding Buffer was added to suspend cells, followed by the addition of 10 µL PI and 5 µL Annexin V-FITC for 15 min at room temperature protected from light (Cell Apoptosis Kit, Becton, Dickinson and Company, New Jersey, USA). Flow cytometry was performed within 1 h. The experiments were performed at least three times independently, with similar results.

### Luciferase assay

Cells were seeded into 24-well plates 24 hours prior to transfection. In the reporter gene experiments, 0.3 μg of either the wild-type or mutant reporter plasmid was co-transfected with 60 nM of control miRNA or miR-183 into the cells in 24-well plates. The transfection was carried out using Lipofectamine 3000 following the manufacturer’s protocol. Subsequently, firefly and Renilla luciferase activities were sequentially measured with the Dual Luciferase Assay System (Promega). The measurements were conducted in strict accordance with the provided guidelines. To account for variations in transfection efficiency, the luciferase activity of firefly was normalized against that of Renilla luciferase expressed from the control vector. Three independent experiments were performed in triplicate.

### Western blot assay

Total cell protein was extracted using RIPA (Beyotime Institute of Biotechnology), and protein concentration was determined using the BCA protein assay kit (Beyotime Institute of Biotechnology). Protein samples were separated by sodium dodecyl sulfate polyacrylamide gel electrophoresis (SDS-PAGE) and transferred to a polyvinylidene fluoride membrane. The membranes were incubated with primary antibody at 4°C overnight on the shaking table

(Ezrin Antibody: CST#3145, RhoA: Affinity#AF6352, RhoC: Affinity#DF6207, ROCK: Affinity#AF7016, β-actin: Proteintech#81115-1-RR). The membranes were then incubated with secondary antibody, followed by chemiluminescence. Gray level was analyzed using the Chemi Capture software.

### Statistical analysis

Data were statistically analyzed using SPSS 21.0 software, and the results are presented as mean ± standard error. When data obeyed normal distribution and homogeneity of variance, one-way analysis of variance with Turkey’s test was used for comparison among multiple groups. If the variance was not uniform, the data were analyzed using Dunnett’s T3 test. We used the Kruskal-Wallis test for non-normally distributed variables. P<0.05 indicated statistical significance.

## Results

### Reduced expression of miR-183 and increased expression of Ezrin in ectopic endometrial stromal cells

**Figure 1:**
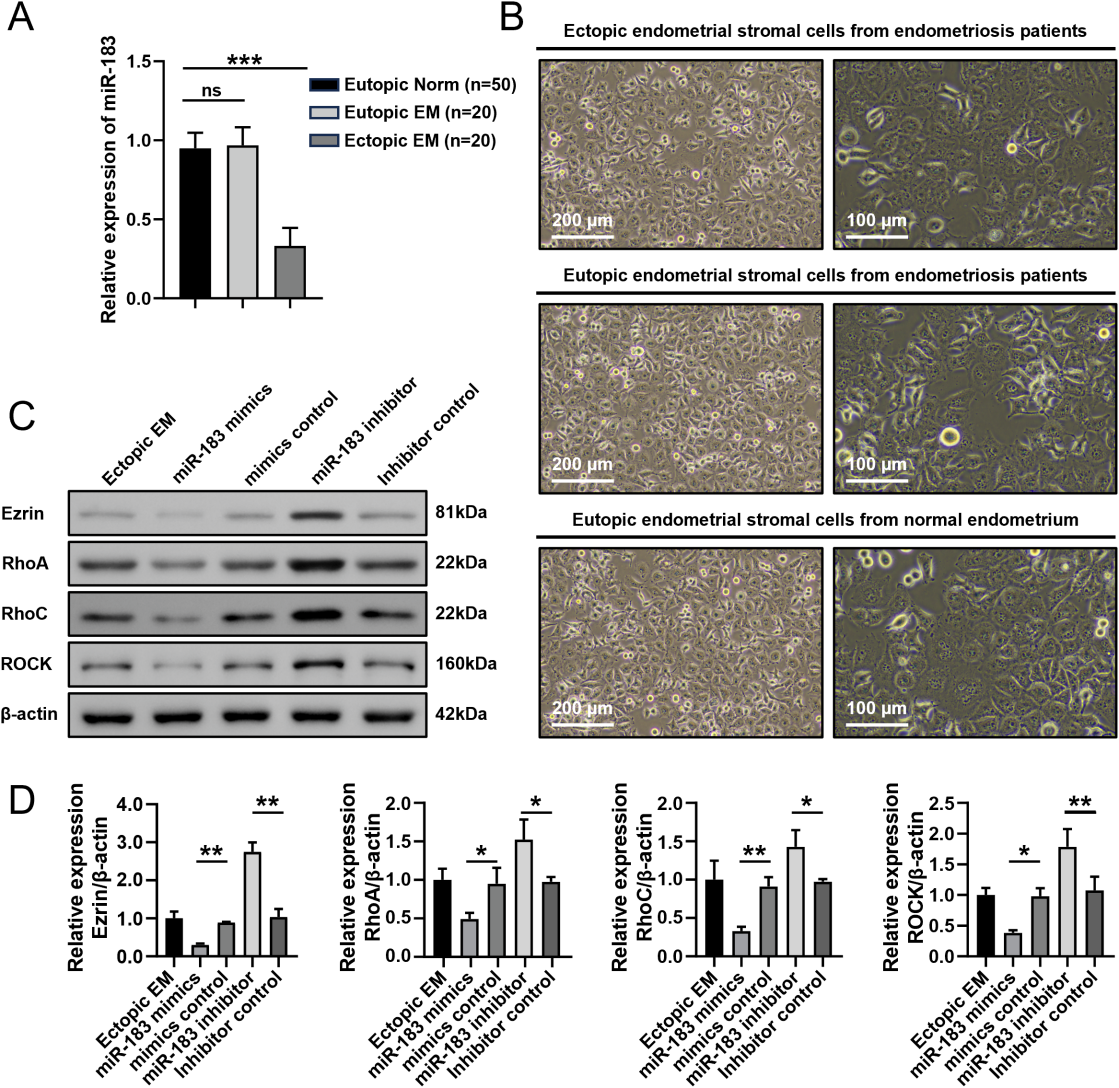
Expression of miR-183 and Ezrin in endometrial stromal cells. **(A)** The expression of miR-183 in endometrial tissue. Data are shown as χ ± s, (Eutopic normal, n=50; Eutopic EM, n=20; Ectopic EM, n=20). Norm, normal endometrium, ***P < 0.001. **(B)** Morphology of ectopic endometrial stromal cells from endometriosis patients, Eutopic endometrial stromal cells from endometriosis patients and Eutopic endometrial stromal cells from normal endometrium (left panel:100×/200 µm, right panel:200×/100 µm). **(C)** Western blot analysis of Ezrin, RhoA, RhoC, and ROCK protein in ectopic endometrial stromal cells. **(D)** Statistical analysis of Ezrin, RhoA, RhoC, and ROCK protein expression in each experimental group. *P < 0.05, **P < 0.01 compared with the control group.

To explore the role of miR-183 in EM, RT-qPCR was first performed to evaluate the level of miR-183 in EM. The results showed that miR-183 expression was markedly reduced in ectopic endometrial tissues compared with normal endometrium tissues (**Figure 1A**). To perform subsequent experiments, we next isolated ESCs from Normal, eutopic and ectopic samples. The endometrial stromal cells adhered to the plate wall; cells showed a polygonal or oval shape with a full and transparent cell body and were in good condition (**Figure 1B**).

We next used western blot to measure the expressions of Ezrin, RhoA, RhoC, and ROCK proteins isolated ESCs transfected with mimics and controls. The protein expression levels of Ezrin, RhoA, RhoC, and ROCK in the miR-183 mimic group were markedly decreased compared with the control (all *P* < 0.01), while miR-183 inhibitor group exhibited the opposite results (**Figures 1C, D**). These results indicated that miR-183 was downregulated in ectopic endometrial tissue and miR-183 mediated regulation of the Rho/ROCK-Ezrin signaling axis.

### Effects of miR-183 on ESC invasion and migration

**Figure 2:**
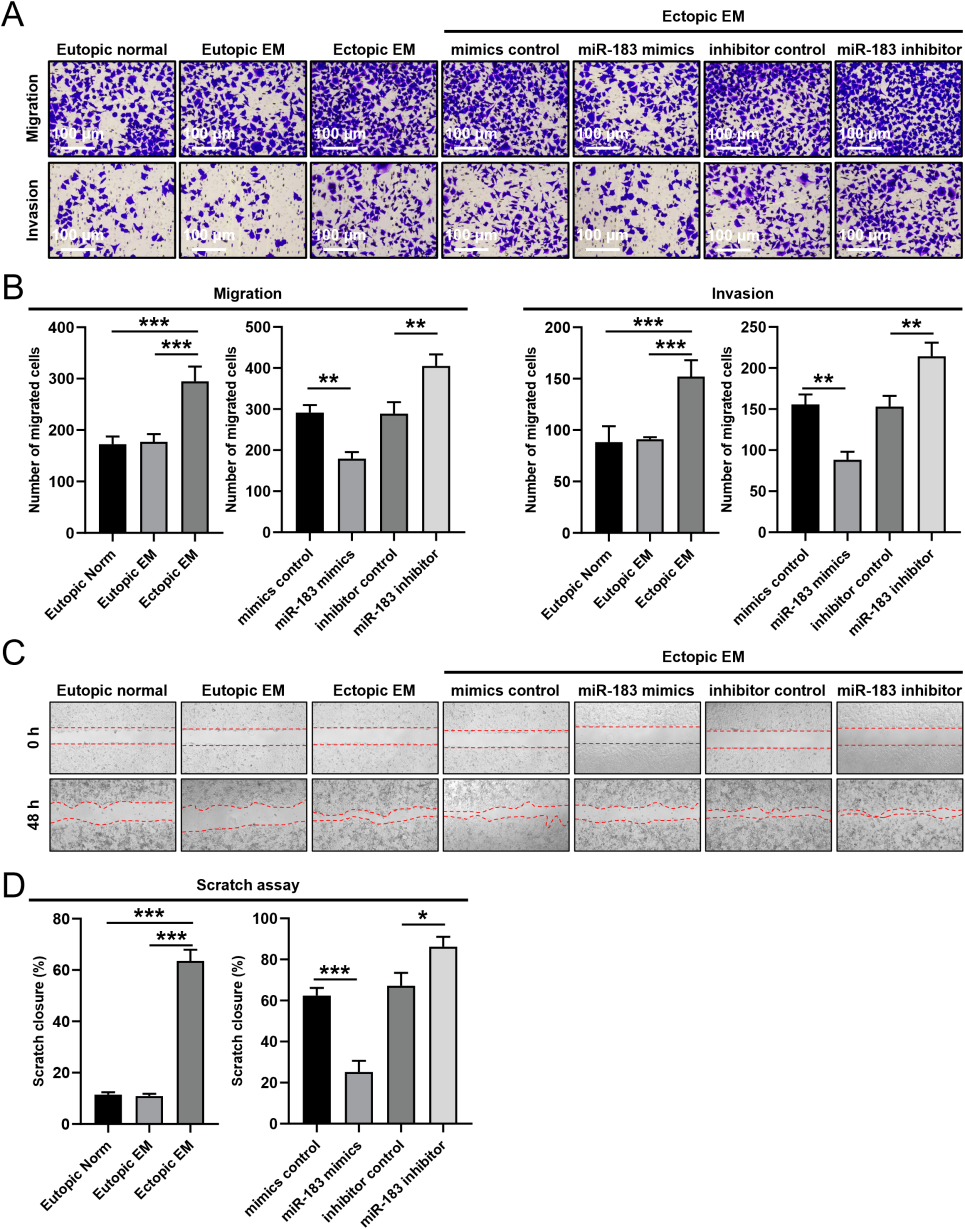
The effect of miR-183 expression on the invasion and migration of endometrial stromal cells. **(A)** The invasion and migration in Eutopic normal, Eutopic EM, and Ectopic EM cells, miR-183 mimics reduced the number of invading and migrating cells, whereas the opposite effect was observed after miR-183 inhibitor (scale bar: 200×/100 µm). **(B)** Data are shown as χ ± s (n=3), **P < 0.01, ***P < 0.001 compared with the control group. **(C)** Cell scratch in Eutopic normal, Eutopic EM, and Ectopic EM cells, miR-183 mimics reduced cell migration ability, whereas the opposite effect was observed after miR-183 inhibitor in cell scratch assay. **(D)** Data are shown as χ ± s (n=3), *P < 0.05, **P < 0.01, ***P < 0.001 compared with the control group.

We conducted Transwell and cell scratch assays on endometrial stromal cells to evaluate the impact of miR-183 on cell invasion and migration. The cell scratch assay quantified cell migration, while the Transwell chamber assessed cell migration and invasion in cells with altered miR-183 expression. Compared with the eutopic normal group and the eutopic endometriosis (EM) group, the invasiveness and migratory capacity of ectopic EM cells were significantly increased (*P* < 0.001). As shown in **Figures 2A, B**, miR-183 mimics transfection markedly reduced the number of invading and migrating cells, whereas the opposite effect was observed after miR-183 inhibitor transfection (*P* < 0.01). A scratch assay was employed to assess cell migration in endometriosis related cells. The scratch closure rate of Ectopic EM cells was significantly higher than that of Eutopic normal and Eutopic EM cells (*P* < 0.001). And miR-183 mimics reduced the scratch closure rate (*P* < 0.001), while the miR-183 inhibitor increased cell migration (*P* < 0.05) (**Figures 2C, D**). These results showed that ectopic EM cells exhibited enhanced migratory ability, and miR-183 overexpression inhibited the migration and invasiveness of ESCs.

### Effects of miR-183 expression on ESC proliferation, apoptosis, and adhesion

**Figure 3:**
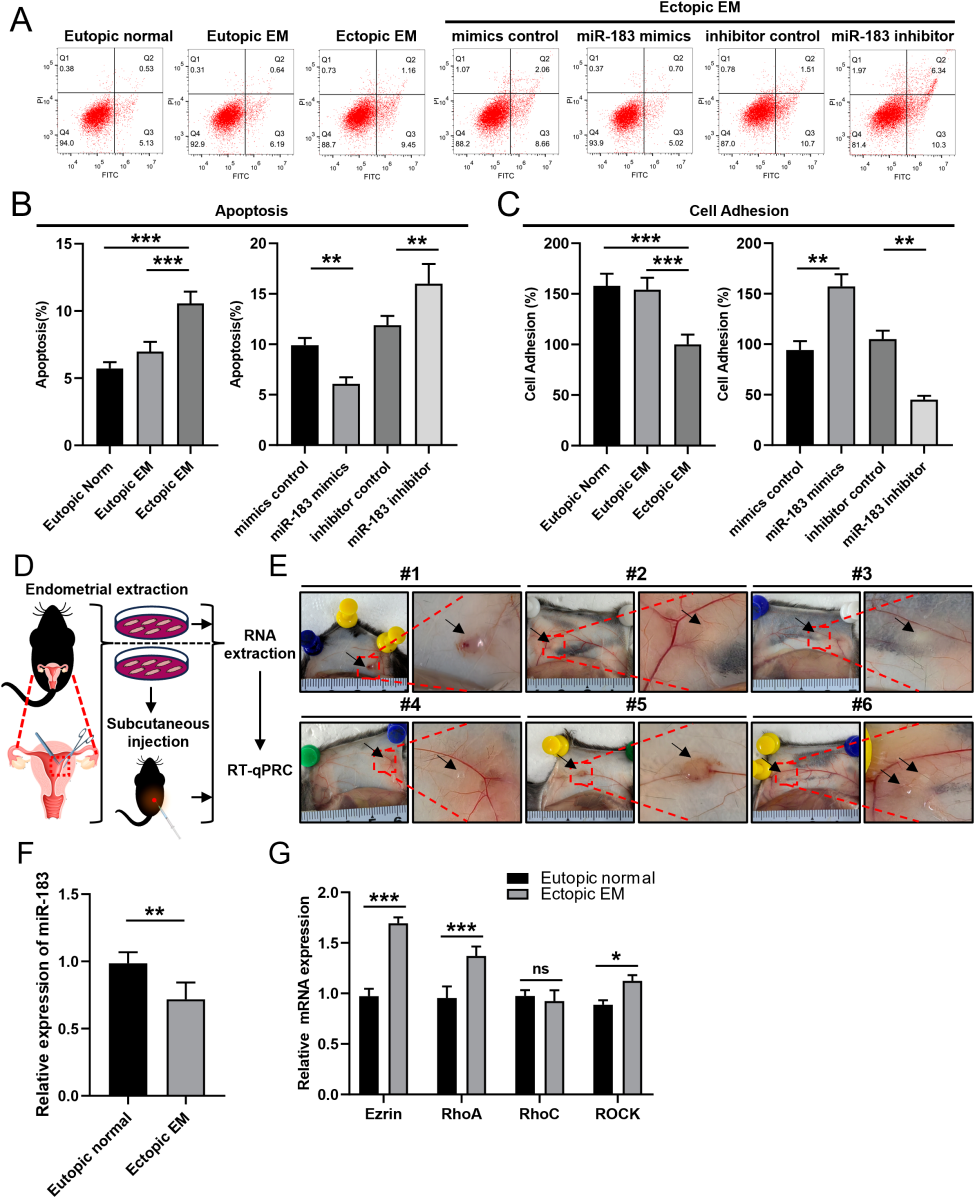
Effects of changes in miR-183 expression on ESC apoptosis and adhesion ability. **(A, B)** Quantification of apoptosis in Eutopic normal cells, Eutopic EM cells, Ectopic EM cells, mimics control group, miR-183 mimics group, inhibitor control group, and miR-183 inhibitor group. **(C)** Quantification of cell adhesion test. Data are shown as χ ± s (n=6), **P < 0.01, ***P < 0.001 compared with the control group. **(D)** Schematic flowchart of the mouse endometriosis model. **(E)** Mouse endometriosis model, the arrows represent endometriosis lesions. **(F)** The expression of miR-183 between endometrial tissue and endometriosis lesions of mice. Data are shown as χ ± s, (n=6), *P < 0.05, **P < 0.01, ns denotes no significant difference (*P* ≥ 0.05). **(G)** mRNA expression of Ezrin, RhoA, RhoC, and ROCK between endometrial tissue and endometriosis lesions of mice.

Overexpression of miR-183 markedly decreased the apoptosis of ESCs (*P* < 0.01), and the apoptotic rate was increased when miR-183 was inhibited (*P* < 0.01) (**Figures 3A, B**). We added more samples for further experiments, while there was no significant difference in the results. CCK-8 assays demonstrated that miR-183 did not markedly alter the cell proliferation rate (**Table 1**). Moreover, miR-183-overexpressing cells showed statistically obvious increases in adhesion (**Figure 3C**). To verify the results *in vivo*, a murine model of endometriosis was established. The surviving endometriotic lesions displayed a variety of morphological characteristics. The majority of the ectopic lesions were cystic, appearing as small blisters with colors ranging from red, white to orange. Some of these cystic lesions were found to be adherent to the surrounding tissues. Additionally, a small proportion of the lesions were solid white in appearance, with no observable blood vessels either on the surface of the lesions or in the surrounding regions (**Figures 3D, E**). RT-qPCR results showed that miR-183 expression was markedly reduced in murine model of endometriosis compared with normal endometrium tissues (**Figure 3F**). The mRNA expression levels of Ezrin, RhoA, and ROCK in the murine model group were markedly increased compared with the normal control (**Figure 3G**). These results indicate miR-183 reduced apoptosis and increases cell adhesion of ESCs but had no effect on cell proliferation.

**Table 1:** Cell proliferation of endometrial stromal cells.

Group	Survival rate
Eutopic normal group	98.54 ± 8.18
Eutopic EM group	96.72 ± 6.81
Blank control group (ectopic EM)	100 ± 6.62
miR-183 mimics group (ectopic EM)	96.66 ± 9.28
mimics control group (ectopic EM)	100.76 ± 7.23
miR-183 inhibitor group (ectopic EM)	102.58 ± 7.52
Inhibitor control group (ectopic EM)	95.82 ± 7.10

### Functional regulation of putative miR-183-binding sites within the 3’UTR of RhoA and Ezrin mRNAs

**Figure 4:**
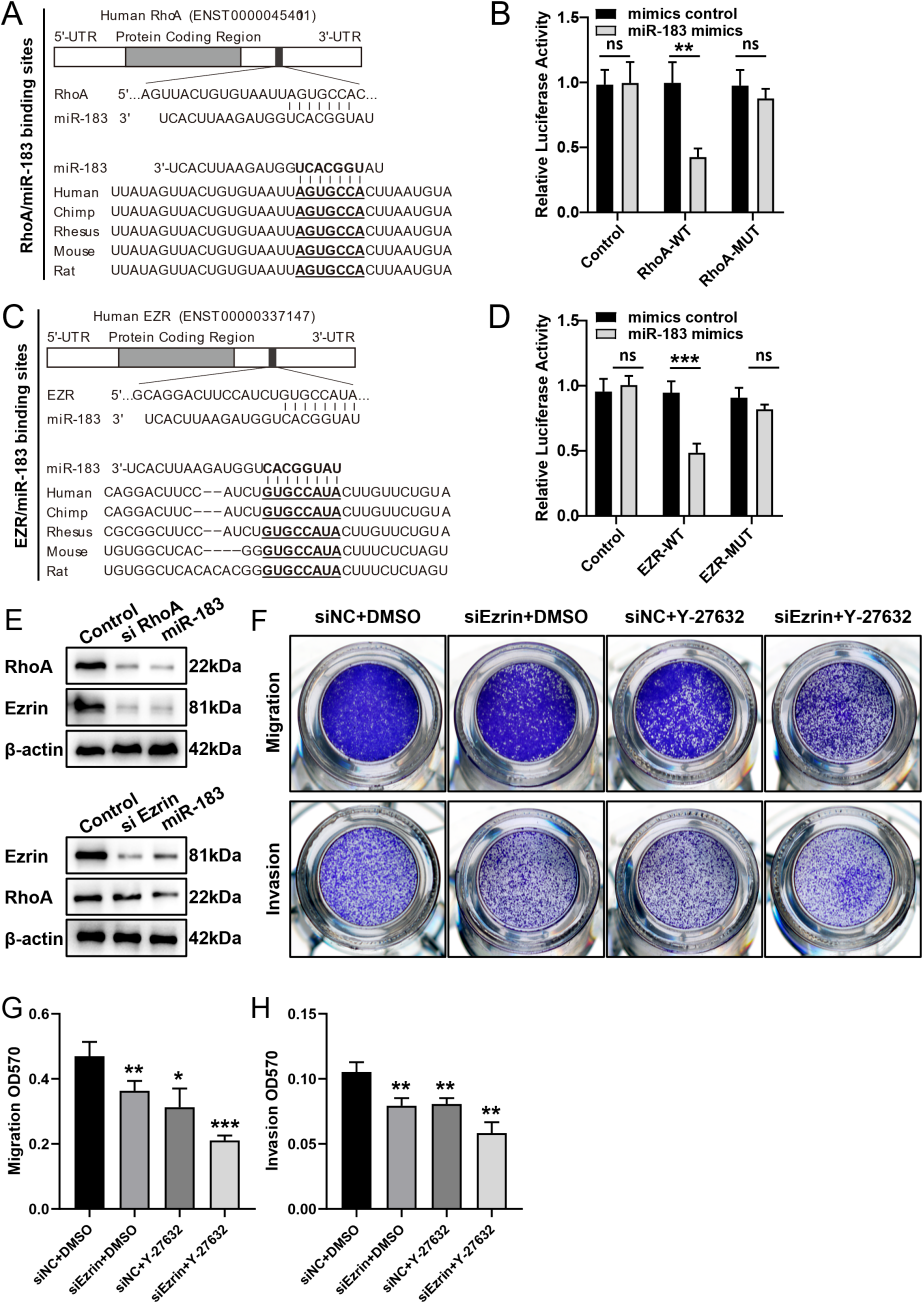
Functional regulation of putative miR-183-binding sites within the 3’UTR of RhoA and Ezrin mRNAs. **(A, C)** Schematic representation of the putative miR-183 target sites in RhoA and Ezrin mRNA. **(B, D)** miR-183 inhibits wild-type but not mutated RhoA and Ezrin 3′UTR reporter activity. **(E)** miR-183 regulates the expression of RhoA and Ezrin. **(F)** Cell migration and invasion assays with si Ezrin alone, ROCK inhibitor (Y-27632) alone and combined si Ezrin +ROCK inhibitor. **(G, H)** Quantification of cell migration and invasion assays, data are shown as χ ± s (n=3), *P < 0.05, **P < 0.01, ***P < 0.001 compared with the control group.

To elucidate the molecular mechanism underlying the inhibitory effect of miR-183, we employed multiple bioinformatics prediction tools, namely PicTar, TargetScan, and miRanda, to identify the potential regulatory targets of miR-183. Through functional classification analysis using the Gene Expression Programming Analysis System (GEPAS), we identified RhoA and Ezrin, two members of the Rho-GTPase family, as promising candidate targets. RhoA and Ezrin are recognized proto-oncogenes, frequently overexpressed in various cancer types, which further underscored their potential importance in the context of miR-183 regulation. Sequence alignment and analysis revealed that the putative miR-183 binding sites were located at nucleotides 739–745 within the 3’UTR of the RhoA mRNA and nucleotides 192–199 within the 3’UTR of the Ezrin mRNA (**Figures 4A, C**). Notably, these nucleotide sequences were highly conserved across multiple species, suggesting evolutionary significance and functional importance.

Given that microRNAs are known to regulate gene expression via base pairing with specific recognition elements within the mRNA of their target genes, a luciferase reporter assay was conducted to validate the specific sequence regions within the 3’UTR of the RhoA and Ezrin mRNA that mediated direct interactions with miR-183. The 3’UTR of the RhoA and Ezrin genes, encompassing the putative miR-183 binding sites, were cloned downstream of the open reading frame of the luciferase gene in the reporter plasmid pGL3. The resulting constructs were designated as pGL3-RhoA-wt and pGL3-Ezrin-wt, respectively. Concurrently, two additional luciferase reporter constructs were engineered, each harboring specific mutations within the predicted miR-183 targeting regions, designed to abrogate miR-183 binding. Upon transient transfection of ectopic EM cells with pGL3-RhoA-wt and miR-183, a significant reduction in luciferase activity was observed when compared to the control group. Furthermore, mutation of the putative miR-183 binding site within the RhoA 3’UTR completely abolished the inhibitory effect on luciferase expression. Analogous results were obtained following co-expression of pGL3-Ezrin-wt or pGL3-Ezrin-mut with miR-183 (**Figures 4B, D**). These findings provide compelling evidence that miR-183 can directly modulate the expression of RhoA and Ezrin by binding to the predicted sequence sites within their respective 3’UTR.

Specific silencing of RhoA using siRNA recapitulated the phenotypic and molecular effects of miR-183 overexpression. RhoA silencing led to a concurrent downregulation of both RhoA and Ezrin protein. In contrast, transfection of cells with siEzrin selectively reduced Ezrin expression without affecting RhoA levels (**Figure 4E**). These findings, consistent with established principles of signal transduction pathways, support the hypothesis that Ezrin functions as a downstream effector within the Rho/ROCK signaling cascade in endometriosis cells.

In ectopic EM cells, rescue experiments were carried out using Ezrin-siRNA, the ROCK inhibitor Y-27632, and their combination. Migration and invasion assays showed that both single treatments significantly inhibited cell migration and invasion, and the combination treatment exerted a more potent inhibitory effect (**Figures 4F–H**). These results suggest that dual targeting of Ezrin and ROCK, although more effective, may not be indispensable for suppressing the motility of ectopic EM cells.

**Figure 5:**
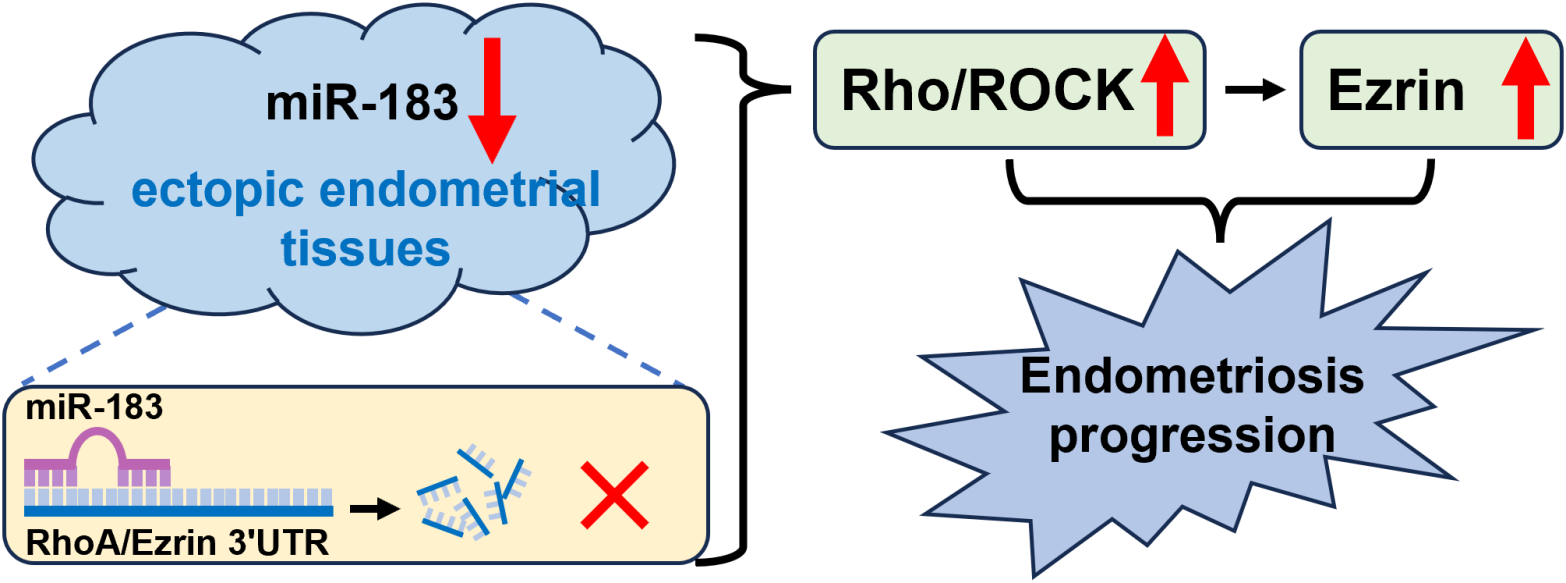
The illustration of miR-183/Rho-ROCK/Ezrin signaling axis in ectopic endometrial tissues. miR-183 directly targets the 3’UTRs of RhoA and Ezrin, downregulating their expression. In endometriosis tissues, reduced miR-183 levels lead to increased RhoA and Ezrin expression. Activated Rho/ROCK signaling further upregulates Ezrin, altering cytoskeletal dynamics and promoting the migration of ectopic endometrial cells.

In summary, miR-183 directly targets the 3’UTRs of RhoA and Ezrin, downregulating their expression. In endometriosis tissues, reduced miR-183 levels lead to increased RhoA and Ezrin expression. Activated Rho/ROCK signaling further upregulates Ezrin, altering cytoskeletal dynamics and promoting the migration of ectopic endometrial cells (**Figure 5**).

## Discussion

EM is a serious reproductive condition among women, defined by the presence of endometrial-like tissues outside of the uterus (38). EM is associated with dysmenorrhea, chronic pelvic pain, infertility, dyspareunia, and some cancers (39). One of the most universal and widely accepted theories of the development of endometriosis is Sampson’s theory of retrograde menstruation (RM), which proposes that RM of endometrial tissue through the fallopian tubes into the peritoneal cavity leads to development and progression of endometriotic lesions (19, 40–42). However, RM is observed in 70%–90% of women and endometriosis only occurs in 7%–10% of women, indicating the role of other factors in the etiology of endometriosis (43, 44). Several studies have found that the occurrence of endometriosis appeared in family aggregations (10). These reports reinforce the potential function of genes in the pathogenesis of endometriosis. Researchers also have found that an altered microenvironment may contribute to the development of endometriosis (11). Additionally, the attachment of the endometrium to the host peritoneum is important for endometriosis progression (12). This attachment is associated with the migration and invasion of endometrial stromal cells, as well as several regulatory factors related to cell migration and invasion, such as miRNAs (17, 24, 26).

Studies have shown that some miRNAs regulate a wide range of target genes to change the biological and physiological characteristics of endometrium (25, 45, 46). MiR-183 plays a key role in the regulation of multiple cellular activities, including invasion, proliferation, and angiogenesis, and it is associated with several malignant biological behaviors. Previous studies showed obvious or mild upregulation of miR-183 in ovarian cancer, colon cancer, lung cancer, breast cancer, laryngeal cancer, endometrial cancer, and osteosarcoma cells (47–52). However, the role of miR-183 in the pathogenesis of EMs has been unclear. In this study, we found that miR-183 is markedly downregulated in ESCs compared with NESCs, suggesting that miR-183 may play a role similar to a tumor suppressor in endometriosis pathogenesis.

While classified as benign, EM exhibits characteristic malignant biological behaviors such as migration, invasion, metastasis, and reoccurrence (14, 53). There is growing evidence that modifications in ESC behaviors, such as proliferation, migration, and invasion, can promote the onset and development of EM (12, 13). Compared with normal endometrial stromal cells from non-endometriotic controls, ectopic endometrial stromal cells from endometriosis demonstrated increased proliferation, migration, and invasion (54). The first step in cell migration and invasion is for cells to change from epithelioid to mesenchymal phenotype (30). Epithelial–mesenchymal transition (EMT), in which epithelial cells become migratory mesenchymal cells, can lead to loss of intercellular adhesion and enhanced migration and invasion (55). In this study, we isolated endometrial stromal cells to investigate the biological function of miR-183. Transwell chamber invasion tests and cell scratch tests showed that miR-183 markedly decreased endometrial stromal cell migration and invasion. Further experiments indicated that overexpression of miR-183 enhanced cell adhesion capacity, while inhibition of miR-183 exhibited the opposite trends. Our findings align with emerging non-invasive diagnostic strategies for endometriosis. Recent advances in liquid biopsy, such as circulating miRNAs and exosome-based biomarkers, complement our identification of miR-183 as a suppressor. While Ronsini et al. highlight the diagnostic potential of liquid biomarkers, our work extends this by elucidating miR-183’s therapeutic mechanism via Ezrin/Rho/ROCK axis inhibition, offering a dual diagnostic and interventional target. This is similar to the findings in previous studies where miR-96-5p,miR-143 and miR-199 exhibited comparable roles in endometriosis, while miR-139, miR-145, and miR-9-5p played opposing roles, suggesting that miR-183 may exert an inhibitory effect in endometriosis (56, 57).

Previous studies have shown that overexpression of miR-183 markedly inhibited cell invasion by downregulation of Ezrin expression (30). But the mechanism of action is still unclear. While Chen et al. linked miR-183 to ITGB1P-mediated invasiveness, we identify Ezrin and Rho/ROCK as critical downstream effectors, broadening understanding of miR-183’s regulatory network. Our findings indicate that while the simultaneous targeting of Ezrin and ROCK yields a more potent inhibitory effect on the motility of ectopic endometriotic (EM) cells, it is not an essential requirement for curbing their migratory and invasive capabilities. In contrast to previous single-target therapeutic strategies, these results present a comprehensive and multi-pronged approach to impeding the progression of endometriosis. This novel perspective may guide the development of more effective treatment regimens that can better address the complex pathophysiology of this disease. By comparing ectopic, non-ectopic, and healthy ESCs, we provide insights into miR-183’s lesion-specific dysregulation, advancing its potential as a biomarker. Ezrin was shown to coprecipitate with two important adhesion molecules, β-catenin and E-cadherin, which further implicates the importance of Ezrin in cell adhesion (30, 58). Upon transfection of ESC cells with the miR-183 mimic, the expression of Ezrin decreased markedly, and transfection with the miR-183 inhibitor exhibited the opposite trend. The expression of miR-183 and Ezrin was negatively correlated in ectopic endometrium tissues of endometriosis patients. We thus speculated that miR-183 may exert its biological function by targeting the degradation of Ezrin. Additionally, the protein expression levels of RhoA, RhoC, and ROCK were markedly reduced in the miR-183 mimic group, indicating that miR-183 markedly inhibited the expression of RhoA, RhoC, and ROCK proteins in endometriosis.

The Rho/Rho-associated protein kinase (Rho/ROCK) signaling pathway plays a crucial role in the regulation of many cellular functions. Rho-associated protein kinase (ROCK) is a 158 kDa serine-threonine kinase. The common upstream activators of ROCK are the Ras superfamily Rho-GTPase proteins RhoA and RhoC, which disrupt the auto-inhibitory function of the carboxyl-terminal region by interacting with the Rho-binding domain during the active, GTP-bound state (59, 60). Upon activation, the Rho subfamily member proteins exert a variety of biological effects by binding to downstream effector molecules (61, 62). Many studies show that ROCK protein plays an important role in tumorigenesis, malignant tumors, cell survival, metastasis, and invasion (62–64). One of the major functions of the Rho/ROCK signaling pathway is to regulate the phosphorylation of myosin-light-chain phosphatase and many other phosphokinases and cytoskeleton-binding proteins (65, 66). Through this mechanism, Rho/ROCK controls cytoskeletal contraction, which is essential for many basic cellular processes, including proliferation, differentiation, apoptosis, and migration. ROCK signaling also regulates the expression and functionality of various cellular miRNAs (67). Previous studies have shown the RhoA-ROCK-mediated pathway contributes to the progression of fibrosis in endometriotic lesions (44). In this study, we found that miR-183 negatively regulates Ezrin expression and ROCK expression, indicating that overexpression of miR-183 inhibits Ezrin expression levels and may markedly inhibit the movement of endometriotic cells through Rho/ROCK signaling.

Because of the similarities between endometriotic cells and cancer cells, the majority of prospective studies on endometriosis are based on cancer research (68). These similarities also suggest that specific signaling molecules and pathways involved in the metastasis and invasion of malignancies may also be related to endometriosis. Abnormalities in pathways such as NF-κB, Hippo/Yes‐associated protein (YAP), MAPK/MEK/ERK, PI3K/Akt/mTOR, Rho/ROCK, Wnt/β‐catenin, and TGF-β pathways may result in ESCs presenting with the tumor-like characteristics of proliferation, autophagy, apoptosis, fibrosis, angiogenesis, ROS production, immune system activation, and inflammatory responses (69). In this study, we found that miR-183 markedly reduced the invasion and migration of endometrial stromal cells and increased cell adhesion. These data suggest that miR-183 may play a role similar to that of a tumor suppressor in endometriosis by targeting Ezrin via the Rho pathway. However, the reduction of miR-183 had no effect on cell proliferation and promoted cell apoptosis, which is inconsistent with the role of tumor suppressors. This may be the difference between endometriosis and malignant tumors or related to different roles of miR-183 in endometriosis through other signaling molecules and pathways. Future study is necessary to clarify this point. This study presents several key strengths that enhance its scientific validity and translational relevance. First, the use of primary human endometrial stromal cells, including ectopic, non-ectopic, and healthy controls, enables direct assessment of miR-183’s lesion-specific role in endometriosis pathogenesis. Second, our multilayered mechanistic validation (e.g., luciferase assays confirming miR-183’s direct binding to Ezrin 3’UTR, siRNA-mediated Ezrin knockdown, and Rho/ROCK pathway inhibition) robustly supports the proposed signaling axis. Third, the translational potential is underscored by functional rescue experiments, which demonstrate that dual targeting of Ezrin and ROCK significantly suppresses cell migration and invasion, suggesting a novel therapeutic strategy. However, several limitations warrant consideration. The absence of *in vivo* validation limits direct extrapolation to clinical settings. While our sample size aligns with similar mechanistic studies, larger cohorts would strengthen statistical power for subgroup analyses. Additionally, the inflammatory microenvironment of endometriosis was not addressed; future work should explore crosstalk between miR-183/Ezrin and cytokines. Finally, while we highlight miR-183’s diagnostic potential, its specificity versus other gynecologic pathologies remains unverified. Despite these constraints, our findings provide foundational insights into miR-183’s role in cytoskeletal regulation and offer actionable targets for future therapeutics.

## Conclusion

Our results show that miR-183, which is expressed in low levels in endometrial of endometriosis patients, may suppress the invasion and migration of endometrial stromal cells in endometriosis patients by targeting Ezrin via the Rho pathway. These findings suggested that miR-183 may represent a potential target for the treatment of endometriosis and provide novel opportunities for clinical therapies for endometriosis.

## Data Availability

The original contributions presented in the study are included in the article/supplementary material. Further inquiries can be directed to the corresponding author.
